# Influence of Ultrasonic and Chemical Pretreatments on Quality Attributes of Dried Pepper (*Capsicum annuum*)

**DOI:** 10.3390/foods12132468

**Published:** 2023-06-23

**Authors:** Milica Lučić, Nebojša Potkonjak, Ivana Sredović Ignjatović, Steva Lević, Zora Dajić-Stevanović, Stefan Kolašinac, Miona Belović, Aleksandra Torbica, Ivan Zlatanović, Vladimir Pavlović, Antonije Onjia

**Affiliations:** 1 Innovation Center of the Faculty of Technology and Metallurgy, Karnegijeva 4, 11120 Belgrade, Serbia; 2 Vinča Institute of Nuclear Sciences–National Institute of Republic of Serbia, University of Belgrade, Mike Petrovića Alasa 12-14, Vinča, 11351 Belgrade, Serbia; 3 Faculty of Agriculture, University of Belgrade, Nemanjina 6, 11080 Belgrade, Serbia; 4 Institute of Food Technology, University of Novi Sad, Bulevar Cara Lazara 1, 21000 Novi Sad, Serbia; 5 Faculty of Mechanical Engineering, University of Belgrade, Kraljice Marije 16, 11120 Belgrade, Serbia; 6 Faculty of Technology and Metallurgy, University of Belgrade, Karnegijeva 4, 11120 Belgrade, Serbia

**Keywords:** Derringer desirability function, factorial design, ultrasound, drying, citric acid, metabisulfite, food quality, antioxidant activity, color

## Abstract

This study investigates the effects of ultrasound, in combination with chemical pretreatments, on the quality attributes (total phenolic and carotenoid content, antioxidant activity (2,2-Diphenyl-1-picrylhydrazyl assay (DPPH)), ferric-reducing ability (FRAP), CIE L* a* b* color, non-enzymatic browning, rehydration ratio, textural and morphological properties) of red pepper subjected to drying (hot air drying or freeze drying). The fractional factorial design was used to assess the impact of factors. The global Derringer desirability function was used to determine the optimal conditions for the best quality attributes of dried pepper. The drying method influenced total phenolic content, a* (redness), and initial rehydration ratio; pretreatment time significantly affected FRAP antiradical activity, a*, chroma and non-browning index, while pH-value had a significant effect on the texture of dried pepper. Non-enzymatic browning was reduced to 72.6%, while the DPPH antioxidant capacity of freeze-dried peppers was enhanced from 4.2% to 71.9%. Ultrasonic pretreatment led to changes in the pepper morphology, while potassium metabisulfite (KMS) was a more effective additive than citric acid.

## 1. Introduction

Pepper (*Capsicum annuum*) is a marketable vegetable and a part of culinary practices worldwide [1]. Peppers are grown on all continents, where China is the largest producer of fresh peppers and India of dried peppers. In 2021, the production of fresh pepper in the world reached 36 million tons, while the production of dried pepper reached 4.8 million tons. China dominates the worldwide production of fresh peppers, with over 16 million tons in 2021. Turkey is in second place with 3.1 million tons, followed by Indonesia (2.7 million tons) and Mexico (2.6 million tons). India is the largest producer of dried pepper (over 2.0 million tons), followed by Thailand (over 0.336 million tons) and China (over 0.312 million tons) (FAOSTAT, 2021) [2]. It is consumed fresh, processed, or in the form of dehydrated products [1,3]. Dehydrated pepper products are whole dried pepper, pepper flakes, and spice [4,5]. Paprika is a non-pungent red pepper powder obtained by grinding dried fruits of different varieties of *C. annuum* [6]. It is used as a colorant and a flavor in preparing different dishes (soups, sauces, stews, processed meats, dairy products, snacks, pizzas, etc.) [1,6,7].

The fruits of fresh peppers are a good source of many compounds with significant antioxidant potential. Antioxidants found in the fruits of sweet *C. annuum* are phenolic compounds, carotenoids, ascorbic acid, capsinoids, vitamin E, and other nutritional components [1,8]. All these nutritional components have a beneficial effect on human health. A diet rich in fruits and vegetables can reduce a person’s risk of developing numerous chronic diseases, such as heart disease, diabetes, cancer, and other diseases [9,10]. Compared with other vegetables, these have the highest amount of vitamin C, carotenoids, and phenolics [11]. In most cases, eating ~60–80 g of fresh pepper is enough to meet the recommended daily intake for vitamin C [10]. Carotenoids are the compounds that are the most accountable for the color (and also influence the price of paprika) of yellow-orange and red varieties [12,13]. While hot capsaicinoid compounds are present in pungent varieties, non-pungent capsinoids are identified in sweet varieties. These compounds also have significant antioxidant properties [14].

Tunnel hot air-drying and sun drying are the most commonly used ways for fruit and vegetable dehydration, although they have certain disadvantages. The main downsides of sun drying are long drying time and risk of fungi proliferation, while high temperature during tunnel drying can result in significant degradation of valuable antioxidant compounds. Furthermore, high-temperature changes the color of the dried product due to oxidation and degradation of carotenoids, as well as the occurrence of Maillard reaction products [6,15,16].

The primary role of food drying is to extend the shelf life of perishable foods [3]. Blanching and chemical pretreatments are often used to decrease drying time, improve color, and better preserve the nutritional components. Ultrasonic pretreatment is one of the possible ways that can be used to produce dried products of better quality [17]. Previous studies indicate that it shortens the drying time and improves the rehydration of mushrooms, Brussels sprouts, and cauliflower [18,19]. Ultrasound pretreatment improves the retention of bioactive compounds and physical properties of fresh-cut quince fruit [20] and the antioxidant properties of ultrasonicated apple–grape juice compared to other treatments [21]. However, data on its influence on the chemical composition and antioxidant properties are scarce. To our knowledge, there is no data on the impact of simultaneous blanching, chemical pretreatments, and ultrasound on dried products. The findings of this study may help to improve the quality of dried peppers and other dried foods. To our knowledge, this is the first study utilizing the experimental design to simultaneously investigate the influence of individual factors (drying and applied pretreatments) and their interactions on the examined quality parameters and overall quality of dried red pepper.

The main goal of this study was to investigate the influence of different processing parameters (the mass of the sample subjected to pretreatment while the volume of the solution is kept constant, pretreatment time, the temperature of the pretreatment solution, application of ultrasound during pretreatment, application of different additives during pretreatment, the impact of pH value, the drying method) and their interactions on physicochemical properties and antioxidant activities of dried sweet red pepper. The fractional factorial design (FFD) was used to study different factors simultaneously. Additionally, the overall quality of dried pepper is studied by combining several responses using the derringer desirability function.

## 2. Materials and Methods

### 2.1. Plant Material, Reagents, and Standards

The fruits of the sweet red peppers (*C. annuum*) cultivar “Horgoš sweet 6” were purchased from a local farmer in Bački Petrovac, Serbia. Trolox (6-hydroxy-2,5,7,8-tetramethylchroman-2-carboxylic acid) and TPTZ (2,4,6-Tris(2-pyridyl)-s-triazine) were purchased from Acros Organics (Fair Lawn, NJ, USA), DPPH (2,2-diphenyl-1-picrylhydrazyl) was purchased from Sigma Aldrich (Darmstadt, Germany), gallic acid and Folin–Ciocalteu’s reagent were purchased from Carlo Erba Reagents S.A.S. (Val de Reuil Cedex, France), HPLC grade methanol was purchased from J.T. Baker (Gliwice, Poland). Citric acid monohydrate, potassium metabisulfite, HCl, acetone, NaOH, FeCl_3_·6H_2_O, sodium carbonate anhydrous (p.a. > 99%), sodium acetate trihydrate (p.a. > 99%) were of analytical grade.

### 2.2. Pretreatment Procedure

Soon after collecting fresh pepper fruits, pretreatments were performed according to the experimental design (Table 1 and Table S1). After the peppers washing, they were cut in half longitudinally and cleaned of seeds, stalks, and placenta. One-half of the experiments were conducted in an ultrasonic bath (Elmasonic S15H, Elma Schmidbauer GmbH, Singen, Germany) at constant power (95 W) and frequency (37 kHz), while another half were done in the same ultrasonic bath but without ultrasonic power (Table S1). The pretreatment solution was filled to the recommended point to achieve uniform ultrasound pretreatment. It is clear that complete uniformity of ultrasound effect is not possible, but sandwich transducer systems, as a part of used ultrasonic bath, enable high homogeneity of ultrasound transmission. Based on the specifications given by the manufacturer, a sweep function was used. According to the manufacturer, the sweep function provides an optimized sound field distribution in the liquid. Additionally, used ultrasound bath unit was equipped with an additional mixing device that assures the optimum mixing of the treated content during the pretreatment. Pretreatment solutions were made with a constant volume of 1 L at mass concentrations of 0.25% citric acid (CA), 0.25% K_2_S_2_O_5_ (KMS), or a mixture of citric acid and K_2_S_2_O_5_ (CA/KMS), each salt having a concentration of 0.25%. Desired pH values for pretreatment solutions were adjusted using 10M NaOH or concentrated HCl.

### 2.3. Drying by Experimental Design

After pretreatments, obtained pepper slices were dried according to the experimental design (Table S1). Tunnel hot air-drying (HD) was carried out as described in the study of Milanović et al. [22] at 60 °C and a constant air velocity of 2 m/s until water content in the final product decreased below 10%. Pepper samples were placed on a perforated tray to allow free circulation of hot air. The relative humidity in the dryer was an unregulated parameter, and its values ranged from 4% to 9%. For freeze-drying (FD) pretreated peppers were frozen at −20 °C and vacuum freeze-dried, maintaining collector temperature at −40 °C and chamber pressure 13.3 Pa for 24 h, using Labconco FreeZone^®^ 18 freeze-dry system (Labconco Corporation, Kansas, MO, USA). The final sample temperature was 25 °C. After dying, all samples were separately vacuum-packed and stored in a dark place at room temperature until analysis. Before analysis, except for the analysis of textural and morphology properties, all samples were grounded to a fine powder. To determine moisture content, the obtained powders were dried at 105 °C to a constant mass [23].

The influence of seven different factors on antioxidant activity, total phenolic content (TPC), total carotenoid content (TCC), the rehydration process, non-enzymatic browning index, surface color, texture and morphological characteristics of dried peppers were assessed using a 1/8 fractional factorial design (FFD) as detailed in Table 1. The design resolution was IV. The alias structure of the FFD is given in Table S2. Five factors were estimated at three levels (−1, 0, +1), and two factors were non-numeric, estimated at two levels: low (−1) and high (+1). Four central points were replicated three times. The experimental design consisted of twenty-eight combinations of seven independent variables (Table S1). Pareto chart, main effect plot, and interaction plot were used to interpret the results. The Pareto chart is a bar chart that ranks the absolute value of the standardized effects of studied factors from the largest to the smallest. The reference line (red line) indicates which effects are statistically significant (in our study level of significance was α = 0.05). Terms A, B, C, D, E, F, and G mark the effect of individual factors, while two or three terms combined denote the effects of factors interactions. The main effect plot shows how factor affects the response. The horizontal line indicates no main effect present. The interaction plot is used to see interactions between factors.

Experimental design allows the study of the influence of several factors simultaneously [24]. Three graphs were used to interpret obtained results: Pareto chart, main effect plot, and interaction plot. Pareto charts provide information about the statistical significance of all variables, where the vertical line is calculated for α = 0.05 and the confidence level 95%. The main effect and interaction plots give additional information about examined variables and their interactions.

### 2.4. Total Phenolic and Carotenoid Analysis

TPC, as gallic acid equivalent, was determined by Folin–Ciocalteu assay according to Dewanto et al. [25] at 760 nm. Approximately 200 ± 1 mg of ground dried pepper was mixed with 5 mL of 80% methanol, sonicated for 30 min at room temperature, and centrifuged for 5 min at 1000× *g*. The supernatant was collected in 10 mL volumetric flask and combined with the next supernatant obtained by re-extraction of the residue under the same conditions. The extraction solution was used to fill up the volumetric flask to the given mark. Every extraction was carried out in triplicate. Obtained extracts were kept at −20 °C until analyses and were analyzed within five days after the extraction. Pepper powders were extracted with acetone until colorless residue and obtained extracts were used to analyze TCC. The absorbance of this solution was measured at 662, 644, and 440 nm [26].

### 2.5. Antioxidant Properties

Antioxidant activity was determined by two assays using the same extracts as for TPC analysis. The results were expressed on dry mass as Trolox equivalent g/kg.

The DPPH assay was done following the method of Thaipong et al. [27] with minor modifications. First, the working solution was prepared by diluting 9 mL of stock solution (25 mg of DPPH in 100 mL of methanol) up to 50 mL with methanol. Then, a reaction mixture was made by mixing 150 µL of extract or standard with 2850 µL of working DPPH solution. The absorbance of the reaction mixture was measured at 517 nm after 30 min incubation at room temperature.

The ferric-reducing ability (FRAP) was assessed using the method of Taipong et al. [27]. The FRAP reagent contained 50 mL of sodium acetate buffer (pH 3.6), 5 mL of 10 mM TPTZ (2,4,6-Tris(2-pyridyl)-s-triazine) solution in 40 mM HCl, and 5 mL of 20 mM FeCl_3_·6H_2_O. The mixture was heated in a water bath to 37 °C and, immediately after that, added to the extract (150 µL of extract or standard + 2850 µL of FRAP reagent). The absorbance of the reaction mixture was measured at 593 nm after 30 min incubation. The buffer solution was used as blank.

### 2.6. Color Analysis

#### 2.6.1. Surface Color Measurement

The color characteristics of powder samples were measured in CIE L* a* b* color space with a Chroma Meter (Model CR-400, Konica Minolta Inc., Tokyo, Japan), using D65 illuminating condition at 2° observed angle. Parameters L* (darkness/whiteness), a* (greenness/redness), b* (blueness/yellowness), chroma C*, and hue angle h* were measured directly. Calibration of the instrument was performed with standard white tile. Three readings were measured for each sample, and an average value was used for data analysis [28].

#### 2.6.2. The Non-Enzymatic Browning Index (NBI)

Extraction of pepper samples was performed according to [29]. The absorbance of the resulting supernatants was measured at 420 nm after four-fold dilution. The results were expressed per kg of dry mass, taking into account the moisture content of the sample (Table S1).

### 2.7. Rehydration Analysis

The pepper flakes were rehydrated in distilled water at 20 ± 1 °C. The solid-to-liquid ratio was 1:50. The rehydration kinetics was followed in time intervals by measuring the mass of slices after 15, 30, 60, 90, 120, 180, 240, 300, 360, 420, and 480 min. Before measuring, the flakes were taken out from the water, drained, and blotted with a paper towel for 20 s. All measurements were carried out in triplicate. The rehydration ratio (RR) was estimated as a ratio of m_t_ to m_0_, where m_t_ is the mass of rehydrated sample at interval t, and m_0_ is the mass of the dried sample before rehydration [30].

### 2.8. Analysis of Textural and Morphological Properties

#### 2.8.1. Texture

The puncture force of dried samples was measured by using a TA.XT Plus Texture Analyser (Stable Micro Systems, Godalming, UK), equipped with a 5 kg load cell. Puncture force was obtained by 1 penetration in each sample (3 strips per treatment), with a 2 mm diameter stainless steel needle probe (P/2N) and a travel distance of 12 mm.

#### 2.8.2. Scanning Electron Microscopy (SEM)

The surface characteristics of dried peppers were analyzed by scanning electron microscope (JEOL JSM6390LV). Sample coating with a layer of Au was performed using a sputter coater (Baltec scd 005) [31].

### 2.9. Desirability Function

The desirability function [32] was used to find the optimal conditions of the examined factors to define the optimal quality levels for different responses. The desirability function is a quick transformation of different responses to one objective function [33]. The desirability function has two steps: (1) transformation of every individual response to an individual desirability function (*d_i_*) that ranges from 0 to 1 (*d_i_* = 0 undesirable response; *d_i_* = 1 desirable response) and (2) calculating of overall desirability (*D*) by taking the geometric average of all individual desirability values (Equation (1)).

(1) $$D={\left(d_{1}^{r_{1}}*d_{2}^{r_{2}}*d_{3}^{r_{3}}*\ldots *d_{n}^{r_{n}}\right)}^{1/\Sigma r_{i}}$$

where *d_i_* is the individual desirability of response *y_i_* (*i* = 1, 2, 3, …, *n*), *n* is the number of responses, and *r_i_* is the importance of every variable relative to others. In our work, we chose weights (*r_i_*) equal to 1 for all twelve responses. The outcome of the overall desirability *D* depends on *r_i_* values that offer users flexibility in the definition of desirability functions. If any of the responses are undesirable, overall desirability will become zero.

Individual desirability is defined by Equation (2) if a response is to be maximized.

(2) $$d_{i}\left(\overset{`}{y_{i}}\left(x\right)\right)=\left[\begin{array}{cc} 0 & if\;{\overset{`}{y}}_{i}\left(x\right)<L_{i}\\ {\left(\frac{{\overset{`}{y}}_{i}\left(x\right)-L_{i}}{U_{i}-L_{i}}\right)}^{s} & if\;L_{i}\leq {\overset{`}{y}}_{i}\left(x\right)\leq U_{i}\\ 1 & if\;{\overset{`}{y}}_{i}\left(x\right)>U_{i} \end{array}\right]$$

Individual desirability is defined by Equation (3) if a response is to be minimized.

(3) $$d_{i}\left(\overset{`}{y_{i}}\left(x\right)\right)=\left[\begin{array}{cc} 1 & if{\overset{`}{y}}_{i}\left(x\right)<L_{i}\\ {\left(\frac{U_{i}-{\overset{`}{y}}_{i}\left(x\right)}{U_{i}-L_{i}}\right)}^{t} & if\;L_{i}\leq {\overset{`}{y}}_{i}\left(x\right)\leq U_{i}\\ 0 & if\;{\overset{`}{y}}_{i}\left(x\right)>U_{i} \end{array}\right]$$

The exponents *s* and *t* are the weights assigned to individual responses that determine how important it is for *d_i_* to be close to maximum or minimum, respectively. In our study, *s* and *t* were chosen to be 1. *U_i_* and *L_i_* are upper and lower acceptable values for the response, respectively [34]. In our study, *L_i_* and *U_i_* are the lowest and the highest values obtained for the response, respectively.

The desired responses for TPC, TCC, antioxidant activity obtained by DPPH and FRAP assays, lightness (L*), redness (a*), chroma (C*), hue (h*), rehydration ratio, texture were set to be maximized, while the desired responses for yellowness (b*) and non-enzymatic browning index were set to be minimized. The importance of all responses was the same.

## 3. Results and Discussion

### 3.1. Total Phenolic Content and Total Carotenoid Content

Among examined parameters, the drying method significantly affected the TPC in pepper (Table S3, Figure S1a). Other parameters and their interactions also influence the TPC, although it is not statistically significant. Tunnel hot air drying was a better method than freeze-drying to preserve TPC (Table S4, Figure S1b). This can be caused by a higher degree of cell destruction throughout hot air-drying at 60 °C compared to freeze-drying, so these compounds are more available for extraction [35] or by the emergence of new phenolic substances due to non-enzymatic interconversion between phenolic molecules [36]. The same trend in TPC was found in control samples produced from fresh pepper (higher TPC in HD than FD samples).

Contrary to this, FD samples produced from water-blanched (WB) pepper had slightly higher TPC than complementary HD samples. For HD samples, all pretreatments had a positive effect compared to water blanching. TPC increases ranged from 2.3% to 91.3%. For FD samples, pretreatments led to both decreases and increases compared to water blanching (from −21.4% to +26.9%). Most of the investigated pretreatments did not contribute to the better preservation of TPC if compared to the control produced from fresh peppers. Slight enhancement in TPC was observed by raising the pretreatment temperature from 20 °C to 50 °C and more intense by increasing the pretreatment time by up to 3 min. A further temperature rise and prolonged pretreatment reduced TPC, probably due to the leaching and degradation of phenolic compounds [37]. The decomposition of phenolic compounds during extraction will likely occur at higher temperatures [37]. The highest retention of TPC was in HD peppers samples with the following pretreatment: pH = 6.5; CA/KMS; without applying ultrasound; T = 50 °C; time = 3 min and mass 100 g in 1 L (experiment 27, Table S1).

This study indicated that most applied pretreatments did not contribute to better preservation of TCC. Control samples revealed that freeze-drying is better for the preservation of TCC (65.45% higher TCC than in HD samples), while water blanching improved the preservation of TCC in the final dried HD sample by 28.64%, but not in FD samples. Contrary to these results, FFD showed that none of the examined parameters have a statistically significant influence on TCC. Additional information on TCC is given in supplementary material S1.

### 3.2. Antioxidant Activity

Two different assays based on radical scavenging capacity (DPPH and FRAP) were used to measure antioxidant activity. The FRAP assay showed higher antioxidant capacity values (from 10.03 ± 1.12 to 20.2 ± 0.03 Trolox equivalent g/kg) compared to the DPPH assay (from 4.0 ± 0.30 to 15.7 ± 0.80 Trolox equivalent g/kg). Also, the FRAP test showed that antioxidant capacity significantly depends on pretreatment time (Table S3, Figure S2a, Pareto chart); for the DPPH test, none of the examined parameters significantly influence antioxidant capacity. Nevertheless, the influence of many individual parameters is the same or similar between these tests (pH value, additive, pretreatment temperature, and mass of treated sample). For FRAP assay, prolonged pretreatment, up to 3 min, enhanced antioxidant capacity (Figure S2b). Further prolongation of pretreatment time reduced antioxidant activity, probably due to the loss of antioxidants that pass into the solution. Both tests indicate that for antioxidant capacity, the best additives were in the following order CA/KSA > KMS > CA. KMS itself acts as an antioxidant and can preserve and stabilize carotenoids that contribute to antioxidant potential [17].

For both tests raising the temperature to 50 °C had a weak positive effect, while higher temperatures led to a sharp decrease in antioxidant capacity. It appears that pretreatment temperature and time had the same effect on TPC (see above). The higher temperatures and prolonged pretreatment caused lower antioxidant capacity due to the leaching and degradation of water-soluble phenolic and other antioxidant compounds responsible for the radical scavenging activity. The reduction of antioxidant capacity during hot water blanching was reported by other authors [38,39]. Our results indicate that the pH value of pretreatment solutions somewhat affected antioxidant capacity (Figure S2a,b). The best results were achieved at a pH value of 6.5.

These two antioxidant tests indicate that activity also depends on certain interactions between parameters. The FRAP assay showed an interaction between the drying method and pretreatment temperature; an interaction between pH and ultrasound pretreatment, while the DPPH assay showed an interaction between the drying method and additive. Better antioxidant activity was achieved at lower pretreatment temperatures, 20 °C and 50 °C for FD samples and 50 °C for HD samples (Figure S2c). Ultrasonic pretreatment positively affected the antioxidant capacity for pH values 3 and 6.5 and had a negative effect at pH value 10 (Figure S2c). The DPPH assay showed that a mixture of CA/KMS was the best additive for both drying methods. Pretreatments with citric acid have yielded the lowest results obtained from antioxidant activity tests when considering HD samples.

Water blanching negatively affected the antioxidant capacity of HD peppers (reduced by 42.2% and 40.9% for DPPH and FRAP assay, respectively) compared to drying without WB. However, WB improved the antioxidant activity of FD peppers for both tests (41.5% and 1.3% for DPPH and FRAP assay, respectively). All pretreatments from the FFD positively affected the ferric reduction ability of HD samples (increments ranged from 5.4% to 105%) compared to WB samples. Our findings are that all of the applied pretreatments from FFD are better for preserving the antioxidant capacity of HD samples than water blanching. Pretreatments from the FFD also improved the DPPH radical scavenging ability of FD samples (increscent ranged from 4.2% to 71.9%) compared to the FD control produced from fresh pepper.

The highest antioxidant capacity, measured by both assays, was obtained for the HD sample with the following pretreatment: pH = 6.5; CA/KMS; applied ultrasound; T = 50 °C; t = 3 min; mass 100 g in 1 L.

### 3.3. Color Analysis

The lightness (L*), yellowness (b*), and hue (h*) of samples were not significantly affected by any of examined parameters. Contrarily, the redness (a*) was found to be significantly dependent on the drying method and the pretreatment time, while the chroma (C*) was significantly affected only by pretreatment time. Better preservation of red pigments was achieved with freeze-drying than tunnel air-drying, probably due to less degradation of red carotenoids (capsanthin and capsorubin) [16], which occurs at higher drying temperatures. A longer pretreatment time positively affected the redness (a*) and color saturation (C*) of paprika. Longer pretreatments, probably due to better absorption of applied additives, gave the final product a more vivid color. In this study, the ultrasound pretreatment did not significantly affect color parameters. The best color characteristic of dried red pepper was obtained for the FD sample with the following pretreatment: pH 3; CA; applied ultrasound; T = 80 °C; t = 5 min; mass 30 g in 1 L.

All pretreatments from FFD, except experiment no. 15, reduce the NBI compared to corresponding controls without pretreatments. Reduction in NBI ranges from 30.7% to 72.6% for HD samples and from 19.6% to 58.5% for FD samples. Non-enzymatic browning was also reduced by water blanching (16.3% and 25.3% in HD and FD samples, respectively). Our results indicate that almost all pretreatments reduced non-enzymatic browning compared to drying without pretreatment. Although longer pretreatments improved the retention of red color, they also significantly affected the browning index due to the formation of brown compounds. Pretreatments with additives were undoubtedly better for reducing non-enzymatic browning than water bleaching. KMS and CA pretreatment solutions enhance the quality of dried foods. Inhibition of non-enzymatic browning with sulfite pretreatment was observed in dried peppers [40], while KMS, CA, and KMS/CA pretreatments improved the color characteristics of sweet bell-pepper powder [41]. In addition, citric acid proved to be a better additive to prevent browning, which was considered safer than a KMS due to some health problems (e.g., asthmatic reactions) [17]. The highest reduction of non-enzymatic browning was obtained for the following pretreatment: pH 3; KMS; applied ultrasound; T = 20 °C; t = 1 min; mass 30 g in 1 L.

### 3.4. Rehydration

**Figure 1:**
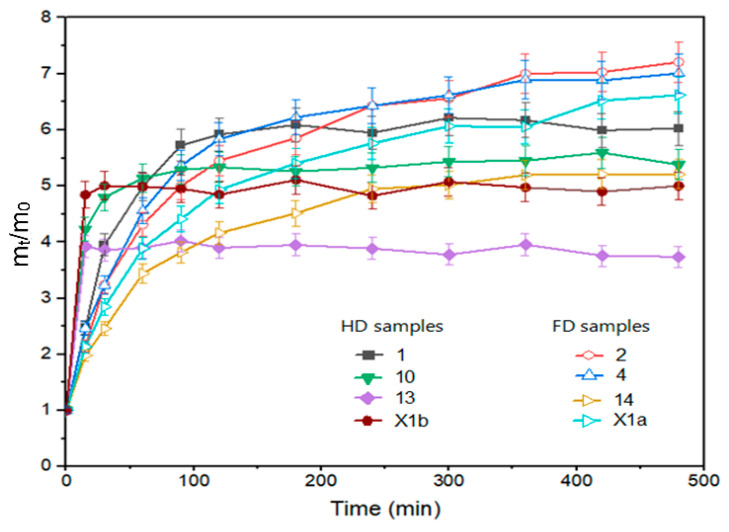
Rehydration ratio of pepper samples, freeze-dried: 1, 10, 13 and hot air-dried: 2, 4, 14 from FFD (Table S1); X1a—hot air-dried control without pretreatment; X1b—freeze-dried control without pretreatment.

The drying method affected the rehydration ratio in the initial period (30 min), where FD samples were rehydrated faster (Figure 1: experiments 1, 10, 13 and control X1b). A higher water absorption rate in the early phase of the rehydration process is observed for freeze-dried peppers compared to hot air-dried peppers (40 °C, 50 °C, 60 °C) by Kheto et al. (2021) [42]. In another study, freeze-dried tomato slices also exhibited higher rehydration ratios during 20 min of rehydration than slices dried using other drying methods [43]. In our study, at the equilibrium point (8 h), the drying method was still the parameter that had the most significant impact on rehydration. However, its impact is not statistically significant and had the opposite effect compared to the onset of rehydration, i.e., samples dried in the air-dryer showed better rehydration (Figure 1 experiment 2, 4, 14 and control X1a). This finding is similar to the results of Kheto et al. (2021) for green, yellow, and red bell peppers [42]. During the rehydration procedure, it was noticed that the HD samples preserved the structure better and that they did not break down during rehydration. FD samples were more brittle after they had been packed into vacuum bags. Fen et al. (2021) [44] and Zheng et al. (2023) [45] reported that during the rehydration of freeze-dried garlic, the potential of water was insufficient to exhaust all intercellular air left behind by the freeze-drying process. This phenomenon may also be the reason for the low final rehydration of freeze-dried pepper samples. Additionally, the vacuum freezing technique can cause structural deformations of the freeze-dried samples [1].

### 3.5. Texture

The pH value of the pretreatment solution had a statistically significant effect on the texture of dried pepper (Table S3, Figure S3a). Higher pH values of the pretreatment solution gave products that are firmer (Figure S3b). This change in the texture is probably induced due to the gelation of the pepper pectin under the influence of monovalent Na^+^ ions, which were added as NaOH to adjust the pH value of the pretreatment solution. Pepper fruits can be a good source of pectin [46]. According to the degree of methylation, there are two groups of pectin, highly methylated pectin (HMP) with a degree of methylation of more than 50% and low methylated pectin (LMP) with a degree of methylation less than 50% [47]. Numerous studies examined the effect of divalent cations (Ca^2+^, Cu^2+^, Fe^2+^) on LMP gelling, but it has been found that monovalent cations can also induce gelling of LMP and HMP [47,48,49]. Alkaline conditions lead to pectin demethylation, after which gel formation can occur under the influence of monovalent cations [49], increasing fruit and vegetable firmness [50]. Wang et al. (2019) [48] found that Na+ and K+ cations in alkaline solutions can lead to HMP gelling, while Pan et al. (2021) [47] found that Na^+^ can lead to LMP gelling.

Other parameters also influence the texture of dried peppers: pretreatment temperature, the interaction between drying and pH value, drying and applied additive, the influence of drying method, and mass of the treated sample (Figure S3c). The main effect plot (Figure S3b) shows that higher temperatures positively affected the texture of dried samples. Blanching processes can activate the enzyme pectin–methylesterase (PME), which de-esterifies pectin, whereby the newly formed product, more precisely its free carboxyl groups, can react with cations present in the solution, resulting in a gelling process [50].

This study also found an interaction between the drying method and pH value and between the drying method and additive. For FD samples, increasing the pH value also increases the strength of the final product, while for HD samples, the best results are achieved at pH 3 and pH 10, where pH 6.5 gives the weakest texture. Moreira et al. (2014) noticed that pectin degradation occurs at pH values of 5.35 and higher, and no gel formation is possible [51]. The pretreatment that gave the firmest peppers was: pH 10; CA; applied ultrasound; T = 80 °C; t = 1 min; mass 170 g in 1 L and hot air-drying.

### 3.6. Morphological Properties of Pretreated Dried Sweet Red Pepper

**Figure 2:**
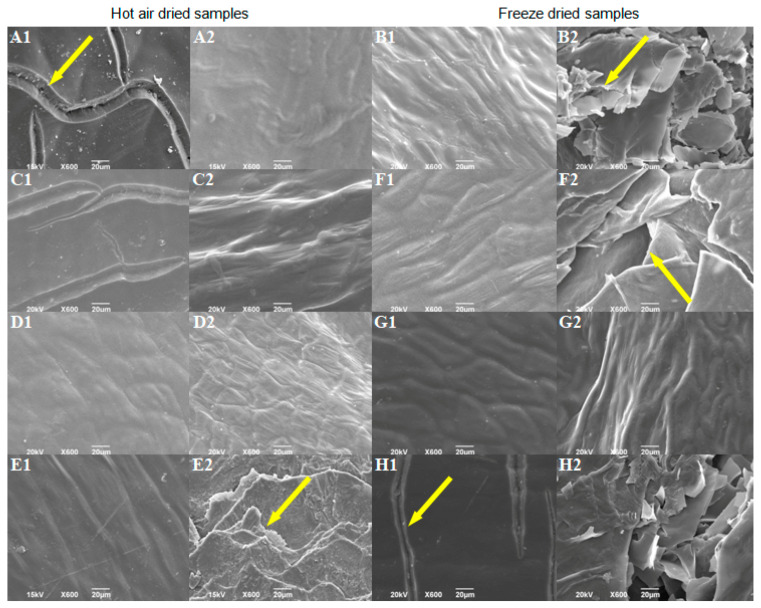
SEM micrographs of the dried sweet pepper: (**A1**)—outer and (**A2**)—the inner surface of HD sample without pretreatment; (**B1**)—outer and (**B2**)—the inner surface of FD sample without pretreatment; (**C1**)—outer and (**C2**)—the inner surface of sample 2 from FFD; (**D1**)—outer and (**D2**)—the inner surface of sample 4 from FFD; (**E1**)—outer and (**E2**)—the inner surface of sample 14 from FFD; (**F1**)—outer and (**F2**)—the inner surface of sample 6 from FFD; (**G1**)—outer and (**G2**)—the inner surface of sample 11 from FFD; (**H1**)—outer and (**H2**)—the inner surface of sample 16 from FFD. Arrows indicate cracks formed during drying.

The SEM analysis was used to examine the influences of different factors on pepper surface properties. Further, the variations in surface properties may indicate potential tissue damage, which could cause the nutritional value to decrease. Morphological properties of controls, without pretreatment, indicate that hot air-drying caused the formation of furrows on the outer surface (Figure 2(A1)), while freeze-drying caused greater changes on the inside of the fruit, i.e., cracking of the inner surface (Figure 2(B2)). Wang et al. [52] also observed similar cracks to those of the hot air-dried control in hot air-dried pepper samples, previously blanched. Higher drying temperatures may result in more damage to the cellular structure [53]. Most pretreatments positively influenced the morphological properties of the dried product compared to controls without pretreatments. Pretreatments reduced (Figure 2(C1)) or completely stopped (Figure 2(D1,E1); Figure S4A,B) the formation of furrows at the outer surface of HD peppers. Vega-Gálvez et al. [54] also observed that sodium metabisulfite pretreated pepper samples (dried at 70 °C) suffered less structural damage than non-pretreated samples. Most of the examined pretreatments did not significantly affect the inner surface of HD peppers (Figure 2(C2,D2); Figure S4C), except the pretreatment No. 14 (Table S1; Figure 2(E2)), which led to considerable cracking of the inner surface compared to the HD control. Changes on the inner surface of dried pepper shown in Figure 2(E2) may result from ultrasonic and temperature (80 °C, 1 min) pretreatment [55,56]. Most of the examined pretreatments did not prevent breakage of the inner surface of freeze-dried samples (Figure 2(F2,H2)), except pretreatment No. 11 (Table S1; Figure 2(G2)). Pretreatment from experiment No. 16 (Table S1; Figure 2(H1,H2)) enhanced the cracking of the outer and inner surfaces. The variation in morphology of pretreated samples may indicate that the ultrasound pretreatment led to changes on the inside surface of pepper fruits (Figure 2(E2,F2)) regardless of whether samples were hot air or freeze-dried.

### 3.7. Overall Desirability

**Figure 3:**
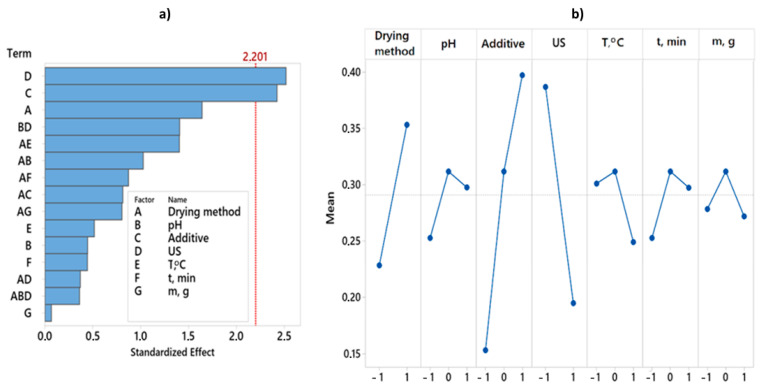
Pareto chart and Main effects plot obtained for overall desirability. (**a**) Pareto chart showing the standardized effect of independent variables and their interactions on overall desirability. (**b**) Main effects plot showing the effect of independent variables. Twelve responses get combined in one desirability function.

**Figure 4:**
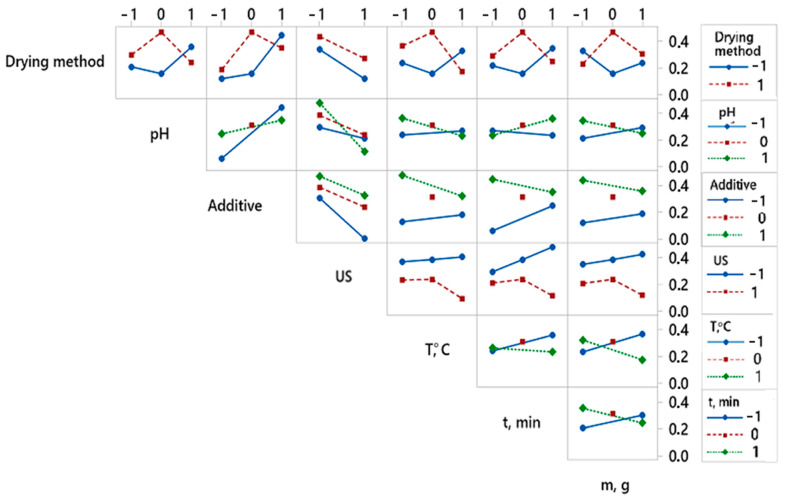
Interaction plot showing the effect of variable’s interactions obtained for overall desirability. Twelve responses get combined in one desirability function.

By analyzing the experimental results with overall desirability function, it was found that the best quality of dried red pepper is significantly influenced by two pretreatment parameters, i.e., ultrasonic pretreatment and type of applied additive (Figure 3). The analysis of variance for the desirability function is presented in Table 2. The better overall quality of dried pepper, when considering 12 responses, is achieved with the following pretreatment: pH value 6.5, KMS pretreatment without applied ultrasound during 3 min at 50 °C, mass to volume ratio 100:1 (g:L), and final freeze-drying (Figure 4). For the overall quality of dried red pepper, ultrasonic pretreatment had a negative effect, probably due to cell wall raptures and the leaching of different bioactive compounds. Applying ultrasound waves in a liquid medium produces cavitations that cause sudden and localized changes in temperature and pressure [57,58]. As a result, bubbles form, rapidly grow, and collapse. When a solid is present in the liquid medium, the acoustic wave can form a microjet in the bubble. This microjet moves through the bubble, leaves it, and passes into the solid, changing the solid structure [1,57,58]. This further leads to the liquid extraction from the solid and the fluid penetration from the outside. The formation of microscopic channels may also occur and facilitate mass transport [1,58]. An interaction plot for 7 observed variables and 12 responses is given in Figure 4.

## 4. Conclusions

The ultrasound and applied additive significantly affected the overall quality of dried red pepper. Regarding the best quality, ultrasound negatively affected physicochemical properties and antioxidant activities, i.e., the overall quality. The best additive was KMS, followed by CA/KMS, while CA exhibited poor results as a pretreatment additive. On the other hand, the drying method, pretreatment time, and pH value significantly influenced individual quality parameters. Hot air-drying provided better results than freeze-drying for retention of TPC and antioxidant capacity while freeze-drying provided better preservation of TCC. The pretreatment time is an important parameter that affected color parameters a*, chroma, and non-enzymatic browning. While prolonged pretreatment positively affected color characteristics, it also influenced higher non-enzymatic browning, even though most of the applied pretreatments reduced non-enzymatic browning (up to 72.6%). The texture was affected by pH value, where higher pH values gave firmer dried peppers.

The relevance of the responses was assumed to be the same; therefore, the same weight was given to each response (equal to 1), which is a limitation of this study. Additional research is required to evaluate the significance of individual responses in the overall quality of dried red pepper.

## Figures and Tables

**Table 1:** Experimental values and coded levels of the factors used for FFD.

No.	Effect of Factor	Factor	Level −1	Level 0	Level +1
1	A	Drying method	HD	-	FD
2	B	pH	3	6.5	10
3	C	Additive (0.25%)	CA	CA/KMS	KMS
4	D	US	Off	-	On
5	E	T (°C)	20	50	80
6	F	t (min)	1	3	5
7	G	m (g)	30	100	170

US—ultrasound; T—pretreatment temperature; t—pretreatment time; m—sample mass; HD—hot air-dried; FD—freeze-dried; CA—citric acid; CA/KMS—citric acid/potassium metabisulfite; KMS—potassium metabisulfite.

**Table 2:** Analysis of variance for desirability function.

Source	DF	Adj SS	Adj MS	F-Value	*p*-Value
Model	16	0.93981	0.058738	1.45	0.271
Linear	7	0.63253	0.090361	2.23	0.114
Drying method	1	0.10933	0.109333	2.69	0.129
pH	1	0.00805	0.008050	0.20	0.665
Additive	1	0.23870	0.238696	5.88	0.034
US	1	0.25751	0.257508	6.34	0.029
T, °C	1	0.01078	0.010783	0.27	0.617
t, min	1	0.00797	0.007974	0.20	0.666
m, g	1	0.00018	0.000181	0.00	0.948
2-Way Interactions	7	0.29276	0.041823	1.03	0.463
Drying method*pH	1	0.04277	0.042775	1.05	0.327
Drying method*Additive	1	0.02684	0.026838	0.66	0.433
Drying method*US	1	0.00554	0.005539	0.14	0.719
Drying method*T, C	1	0.08010	0.080101	1.97	0.188
Drying method*t, min	1	0.03090	0.030902	0.76	0.402
Drying method*m, g	1	0.02626	0.026262	0.65	0.438
pH*US	1	0.08034	0.080342	1.98	0.187
3-Way Interactions	1	0.00530	0.005301	0.13	0.725
Drying method*pH*US	1	0.00530	0.005301	0.13	0.725
Curvature	1	0.00922	0.009221	0.23	0.643
Error	11	0.44673	0.040611		
Lack-of-Fit	3	0.28896	0.096321	4.88	0.032
Pure Error	8	0.15776	0.019720		
Total	27	1.38653			

*—indicate interactions between factors.

## Data Availability

The data that support the findings of this study are available from the corresponding authors upon reasonable request.

## Supplementary Materials

The following supporting information can be downloaded at: https://www.mdpi.com/article/10.3390/foods12132468/s1, Figure S1: Total phenolic content (TPC): (a) Pareto chart of standardized effect (response in g/kg GAE, α = 0.05); (b) Main effect plot for g/kg GAE; (c) Interaction plot for g/kg GAE; Figure S2: FRAP assay: (a) Pareto chart of standardized effect (response in equivalent Trolox g/kg, α = 0.05); (b) Main effect plot for equivalent Trolox g/kg ; (c) Interaction plot equivalent Trolox g/kg; Figure S3: Texture analysis / Skin puncture force (g): (a) Pareto chart for standardized effect; (b) Main effect plot; (c) Interaction plot; Figure S4: SEM micrographs of the dried sweet pepper: (A) outer surface of sample 3 from FFD, (B) outer surface of sample 24 from FFD, (C) inner surface of sample 24 from FFD; Table S1: Experimental values for the fractional factorial design (FFD) and results obtained for all the independent variables; Table S2: Alias Structure for 1/8 FFD, 7 factors, 28 runs, resolution IV; Table S3: Main and interaction effects for each of the independent variables.; Table S4: Results for control pepper samples g/kg dry basis [59,60].
