# Supplementary material for: Influence of Ultrasonic and Chemical Pretreatments on Quality Attributes of Dried Pepper (Capsicum annuum)
Source: Foods. 2023 Jun 23;12(13):2468. doi: 10.3390/foods12132468 (PMC10340434; doi:10.3390/foods12132468)
Supplement: Supplementary file 1 [file foods-12-02468-s001.zip › foods-2431217-supplementary.pdf]

foods MDPI

Influence of Ultrasonic and Chemical Pretreatments on Quality Attributes of Dried Pepper (*Capsicum annuum*)

Milica Lučić [1], Nebojša Potkonjak [2,*], Ivana Sredović Ignjatović [3], Steva Lević [3], Zora Dajić-Stevanović [3], Stefan Kolašinac [3], Miona Belović [4], Aleksandra Torbica [4], Ivan Zlatanović [5], Vladimir Pavlović [3] and Antonije Onjia [6,*]

1 Innovation Center of the Faculty of Technology and Metallurgy, Karnegijeva 4, 11120 Belgrade, Serbia
2 Vinča Institute of Nuclear Sciences—National Institute of Republic of Serbia, University of Belgrade, Mike Petrovića Alasa 12-14, 11351, Vinča, Belgrade, Serbia
3 Faculty of Agriculture, University of Belgrade, Nemanjina 6, 11080 Belgrade, Serbia
4 Institute of Food Technology, University of Novi Sad, Bulevar Cara Lazara 1, 21000 Novi Sad, Serbia
5 Faculty of Mechanical Engineering, University of Belgrade, Kraljice Marije 16, 11120 Belgrade, Serbia
6 Faculty of Technology and Metallurgy, University of Belgrade, Karnegijeva 4, 11120 Belgrade, Serbia
* Correspondence: npotkonjak@vin.bg.ac.rs (N.P.); onjia@tmf.bg.ac.rs (A.O.)

Supplementary Material S1

Although none of the examined parameters have a statistically significant influence on TCC, it is observed the certain impact of (1) interaction between the drying method and mass-to-volume ratio; (2) drying method; (3) interaction between drying method and additive; (4) mass to volume ratio. Generally, tunnel hot air-drying more negatively affected TCC compared to freeze-drying. This finding is consistent with Kaur et al. (2020) [59], who observed more losses of carotenoids at 60 °C compared to lower drying temperatures. Treatments from the design lead to both a reduction and an increase in TCC compared to controls (HD and FD controls) produced from WB pepper. The change in TCC ranged from -38.67% to +18.30% in HD samples and from -39.42% to +5.03% in FD samples. The highest retention of TCC was in the FD sample with the following pretreatment: pH=10; CA; ultrasonic applied; T=20 °C; t=1 min and mass 30 g in 1 L. For the HD sample, the best TCC retention was obtained for pretreatment with pH=6.5; CA/KMS; ultrasonic applied; T=50 °C; t=3min and mass 100 g in 1 L. The positive impact of CA/NaMS (citric acid/sodium metabisulfite) on TCC was also observed by Bechoff et al. [60].

Table S1. Experimental values for the fractional factorial design (FFD) and results obtained for all the independent variables.

No.	Drying Method	pH	Additive	Ultrasonic power	T (ºC)	t (min)	Mass (g)	Moisture content (%)	TPC mg/g GAE	DPPH	L*	a*	b*	C*	h*	TCC µg/g	NBI	FRAP	Skin puncture force (g)	RR
1	FD	3	KMS	ON	20	1	170	5.22	8.69	44.67	47.90	31.31	32.08	44.83	45.69	3428	0.4487	65.23	157.33	6.0256
2	HD	6.5	CA/ KMS	OFF	50	3	100	8.12	9.40	40.58	43.17	27.21	26.54	38.00	44.29	3303	0.9313	52.75	83.60	7.2103
3	HD	6.5	CA/ KMS	ON	50	3	100	8.77	8.74	36.93	42.61	27.59	24.11	36.63	41.15	4046	0.5279	52.63	208.30	6.1286
4	HD	10	KMS	OFF	20	1	170	10.22	11.33	49.48	45.08	28.80	27.92	40.10	44.11	4365	0.6453	58.46	190.67	7.0100
5	FD	10	CA	OFF	20	5	170	7.90	10.09	40.07	44.97	31.87	29.40	43.36	42.70	4363	0.6986	66.31	205.08	5.9075
6	FD	6.5	CA/ KMS	ON	50	3	100	7.68	8.87	42.07	43.85	31.84	27.99	42.39	41.32	5472	0.4164	67.41	185.92	5.8904
7	HD	3	KMS	OFF	80	5	170	6.22	9.44	42.13	43.07	30.85	27.58	41.38	41.79	4816	0.3953	53.38	250.92	5.7331
8	HD	6.5	CA/ KMS	ON	50	3	100	7.76	10.41	45.84	40.89	30.64	25.65	39.96	39.93	584	0.9055	65.22	219.36	5.7814
9	FD	10	KMS	OFF	80	1	30	6.59	8.71	28.69	43.16	33.89	30.35	45.49	41.85	5484	0.5279	50.56	238.34	5.6819
10	FD	6.5	CA/ KMS	ON	50	3	100	6.94	11.37	40.81	43.20	31.92	30.85	44.39	44.02	4068	0.7663	68.21	134.95	5.3787
11	FD	6.5	CA/ KMS	OFF	50	3	100	9.14	8.68	33.63	48.91	30.84	31.96	44.41	46.02	4099	0.7245	62.07	167.44	6.8664
12	FD	3	CA	OFF	80	1	170	4.04	7.16	27.06	47.04	29.44	32.96	44.19	48.23	3925	0.6629	40.40	159.46	5.8043
13	FD	10	CA	ON	20	1	30	4.16	9.35	43.11	44.60	31.95	30.98	44.51	44.12	5943	0.4030	60.85	187.40	3.7370
14	HD	10	CA	ON	80	1	170	4.28	7.94	16.03	45.67	27.95	31.61	42.19	48.51	3179	0.5123	40.07	257.18	5.2077
15	FD	3	CA	ON	80	5	30	7.39	8.09	29.09	48.26	36.95	36.22	51.75	44.43	5093	1.1605	50.15	116.97	5.2348
16	FD	3	KMS	OFF	20	5	30	3.70	8.96	36.91	48.49	33.36	33.09	46.98	44.77	4732	0.6702	69.47	108.04	5.7237
17	HD	6.5	CA/ KMS	OFF	50	3	100	3.45	9.63	33.41	43.79	30.14	29.70	42.31	44.58	4919	0.9998	62.14	179.60	5.4375
18	FD	6.5	CA/ KMS	OFF	50	3	100	*6.94*	8.71	41.44	49.54	32.27	33.87	46.78	46.36	4084	0.7526	62.15	168.95	5.6108
19	FD	6.5	CA/ KMS	ON	50	3	100	*7.31*	10.12	37.25	43.52	31.88	29.42	43.39	42.67	4770	0.5913	68.34	160.43	5.6345
20	FD	10	KMS	ON	80	5	170	3.11	7.04	33.24	48.80	33.18	33.45	47.11	45.23	4515	0.5902	57.36	222.55	5.4940
21	HD	3	KMS	ON	80	1	30	4.24	9.00	26.06	44.33	29.45	29.85	41.94	45.38	3831	0.5193	51.52	153.02	5.2249
22	FD	6.5	CA/ KMS	OFF	50	3	100	4.75	8.73	40.88	50.17	33.71	35.78	49.16	46.71	4069	0.7803	62.24	170.47	4.3552
23	HD	10	KMS	ON	20	5	30	6.46	10.32	33.92	46.86	30.23	32.04	44.05	46.67	3470	0.6790	64.84	161.80	6.0290
24	HD	3	CA	ON	20	5	170	11.06	12.26	23.38	45.91	31.30	34.02	46.22	47.38	3028	0.8317	57.91	182.93	7.1410
25	HD	10	CA	OFF	80	5	30	5.41	11.14	24.82	47.19	31.88	33.37	46.15	46.30	3737	0.9791	71.33	257.16	6.3561
26	HD	3	CA	OFF	20	1	30	6.68	7.52	15.82	45.16	30.81	32.40	44.72	46.44	3313	0.4641	43.43	214.92	6.4187
27	HD	6.5	CA/ KMS	OFF	50	3	100	10.07	14.06	27.57	48.21	33.28	35.46	48.63	46.82	4129	0.5708	79.13	153.80	6.2203
28	HD	6.5	CA/ KMS	ON	50	3	100	5.62	12.48	62.85	40.89	30.64	25.65	39.96	39.93	5339	0.7231	80.55	147.72	5.4539

Table S2. Alias Structure for 1/8 FFD, 7 factors, 28 runs, resolution IV.

**Alias Structure**
I + ABCE + ABFG + ACDG + ADEF + BCDF + BDEG + CEFG
A + BCE + BFG + CDG + DEF + ABCDF + ABDEG + ACEFG
B + ACE + AFG + CDF + DEG + ABCDG + ABDEF + BCEFG
C + ABE + ADG + BDF + EFG + ABCFG + ACDEF + BCDEG
D + ACG + AEF + BCF + BEG + ABCDE + ABDFG + CDEFG
E + ABC + ADF + BDG + CFG + ABEFG + ACDEG + BCDEF
F + ABG + ADE + BCD + CEG + ABCEF + ACDFG + BDEFG
G + ABF + ACD + BDE + CEF + ABCEG + ADEFG + BCDFG
AB + CE + FG + ACDF + ADEG + BCDG + BDEF + ABCEFG
AC + BE + DG + ABDF + AEFG + BCFG + CDEF + ABCDEG
AD + CG + EF + ABCF + ABEG + BCDE + BDFG + ACDEFG
AE + BC + DF + ABDG + ACFG + BEFG + CDEG + ABCDEF
AF + BG + DE + ABCD + ACEG + BCEF + CDFG + ABDEFG
AG + BF + CD + ABDE + ACEF + BCEG + DEFG + ABCDFG
BD + CF + EG + ABCG + ABEF + ACDE + ADFG + BCDEFG
ABD + ACF + AEG + BCG + BEF + CDE + DFG + ABCDEFG

Terms A, B, C, D, E, F, and G denotes effects of analyzed factors on responses, i.e. A – effect of drying method; B – effect of pH values of pretreatment solution; C – effect of applied additive; D – effect of ultrasound pretreatment; E – effect of pretreatment temperature, °C; F – effect of pretreatment time, min; and G – effect of sample mass (g) that is treated during a pretreatment. Design Generators: E = ABC; F = BCD; G = ACD.

Table S3. Main and interaction effects for each of the independent variables.

TPC	A*	BD	E	F	AF	ABD	AG	B	AD	AC	AE	G	D	AB	C
TCC	AG	A	AC	G	AB	B	C	D	E	AD	ABD	AE	BD	AF	F
DPPH	C	AC	E	G	AE	A	D	B	AG	AF	BD	ABD	F	AB	AD
FRAP	F*	E	AE	BD	C	B	ABD	AF	AD	G	AG	AB	A	D	AC
L*	A	AB	D	F	AC	AG	ABD	AF	AE	B	BD	AD	E	C	G
a*	A*	F*	G	BD	AG	AD	AE	ABD	AC	E	B	AB	AF	D	C
b*	A	C	AC	D	AE	AB	F	G	B	ABD	AG	E	AD	BD	AF
C*	A*	F	G	AC	AE	C	D	B	AD	AB	E	BD	AG	AF	ABD
h*	AB	C	D	AG	AC	BD	ABD	AE	F	B	G	AD	A	AF	E
NBI	F*	BD	AB	C	AE	G	D	E	ABD	A	AF	AC	B	AD	AG
Skin puncture force	B*	E	AB	AC	A	G	ABD	AF	C	AE	BD	F	D	AD	AG
RR	A	AE	D	BD	G	AC	E	F	AB	B	AG	C	AD	AF	ABD

Terms A, B, C, D, E, F, and G denotes effects of analyzed factors on responses, i.e. A – effect of drying method; B – effect of pH values of pretreatment solution; C – effect of applied additive; D – effect of ultrasound pretreatment; E – effect of pretreatment temperature, °C; F – effect of pretreatment time, min; and G – effect of sample mass (g) that is treated during a pretreatment. The effect of interactions between two or three factors is denoted with two or three capital letters. The effects are ranked according to their impact on the examined variable, from the largest to the smallest. Statistically significant effects (α=0.05) have * in subscript.

Table S4. Results for control pepper samples g/kg dry basis.

**Control**	**Pretreatment**	**Drying method**	**DPPH[a]**	**FRAP[a]**	**TPC[b]**	**TCC**
X1a	-	HD	7.53±0.80	16.3±1.57	11.6±1.46	3.84±0.42
X1b	-	FD	6.51±1.22	13.2±1.93	8.46±1.31	5.86±0.21
X2a	Blanched[#]	HD	5.66±0.43	9.66±0.03	7.35±0.39	4.91±0.33
X2b	Blanched[#]	FD	9.21±0.31	13.4 ±0.03	8.96±0.58	5.60±0.47

[#]water blanched at 80 °C, 3 minutes; [a] - Trolox equivalent g/kg; [b] - GAE equivalent g/kg, GAE –gallic acid.

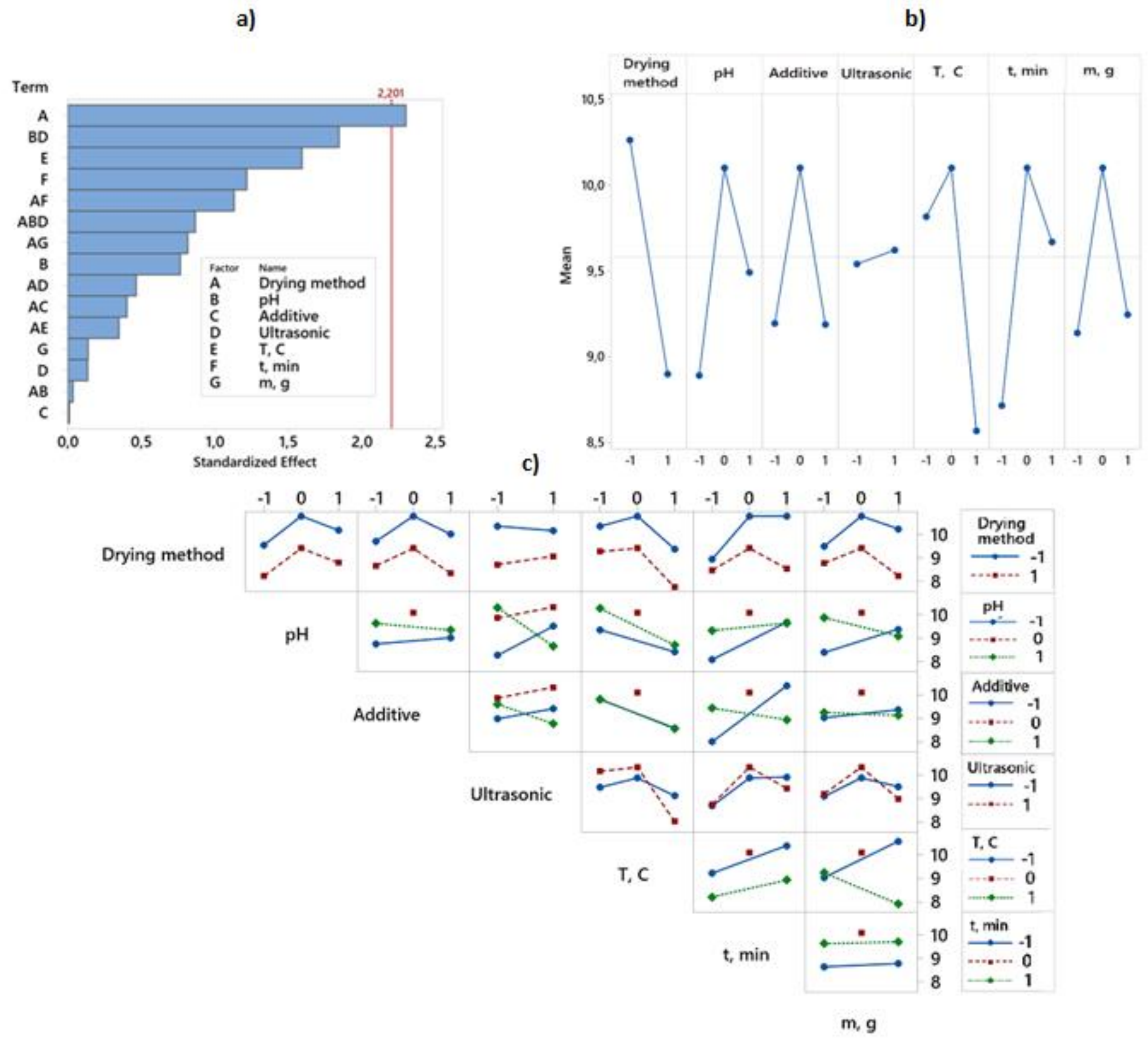

Figure S1. Total phenolic content (TPC): (**a**) Pareto chart of standardized effect (response in g/kg GAE, α=0.05); (**b**) Main effect plot for g/kg GAE; (**c**) Interaction plot for g/kg GAE.

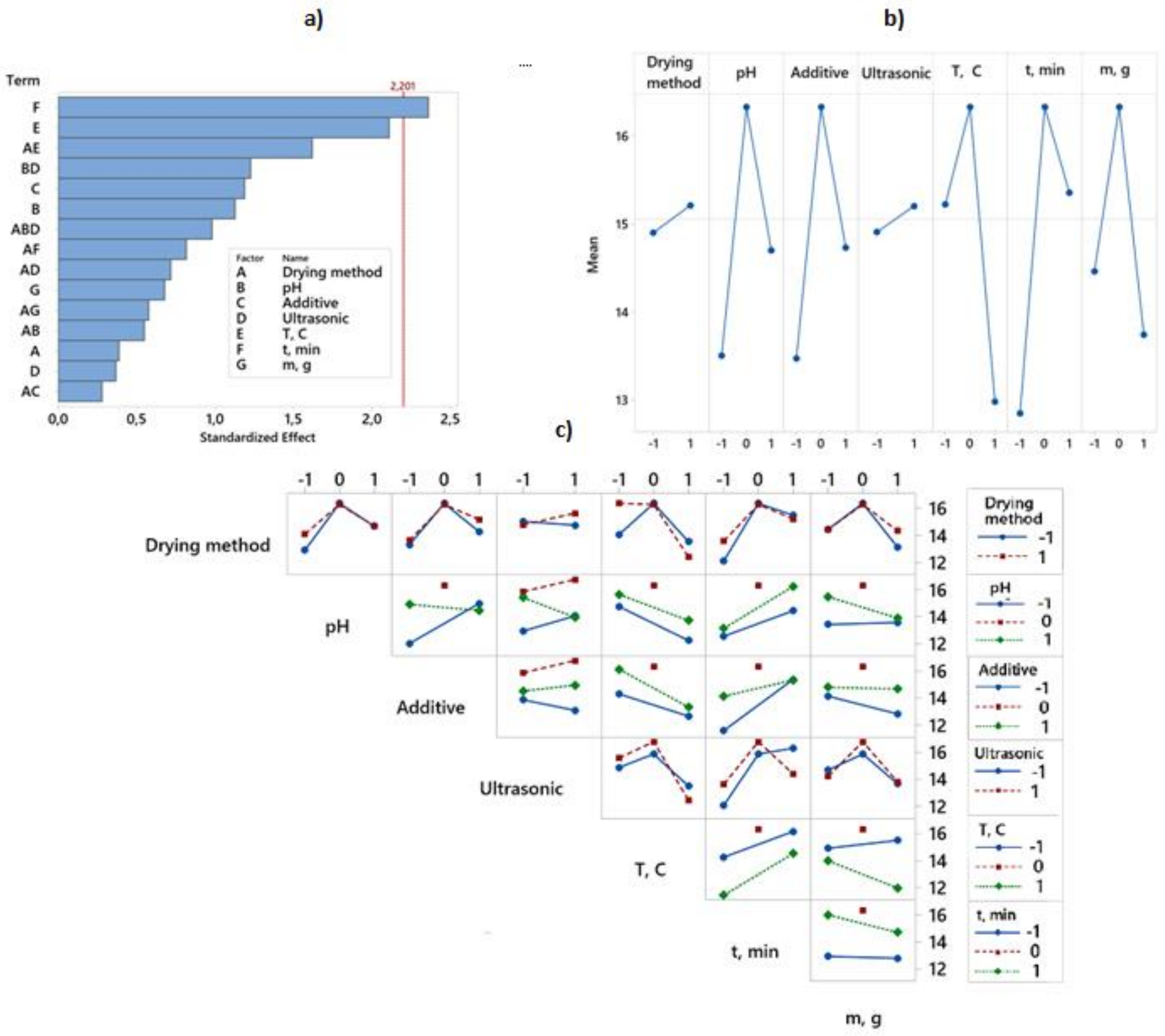

Figure S2. FRAP assay: (**a**) Pareto chart of standardized effect (response in equivalent Trolox g/kg, α=0.05); (**b**) Main effect plot for equivalent Trolox g/kg ; (**c**) Interaction plot equivalent Trolox g/kg.

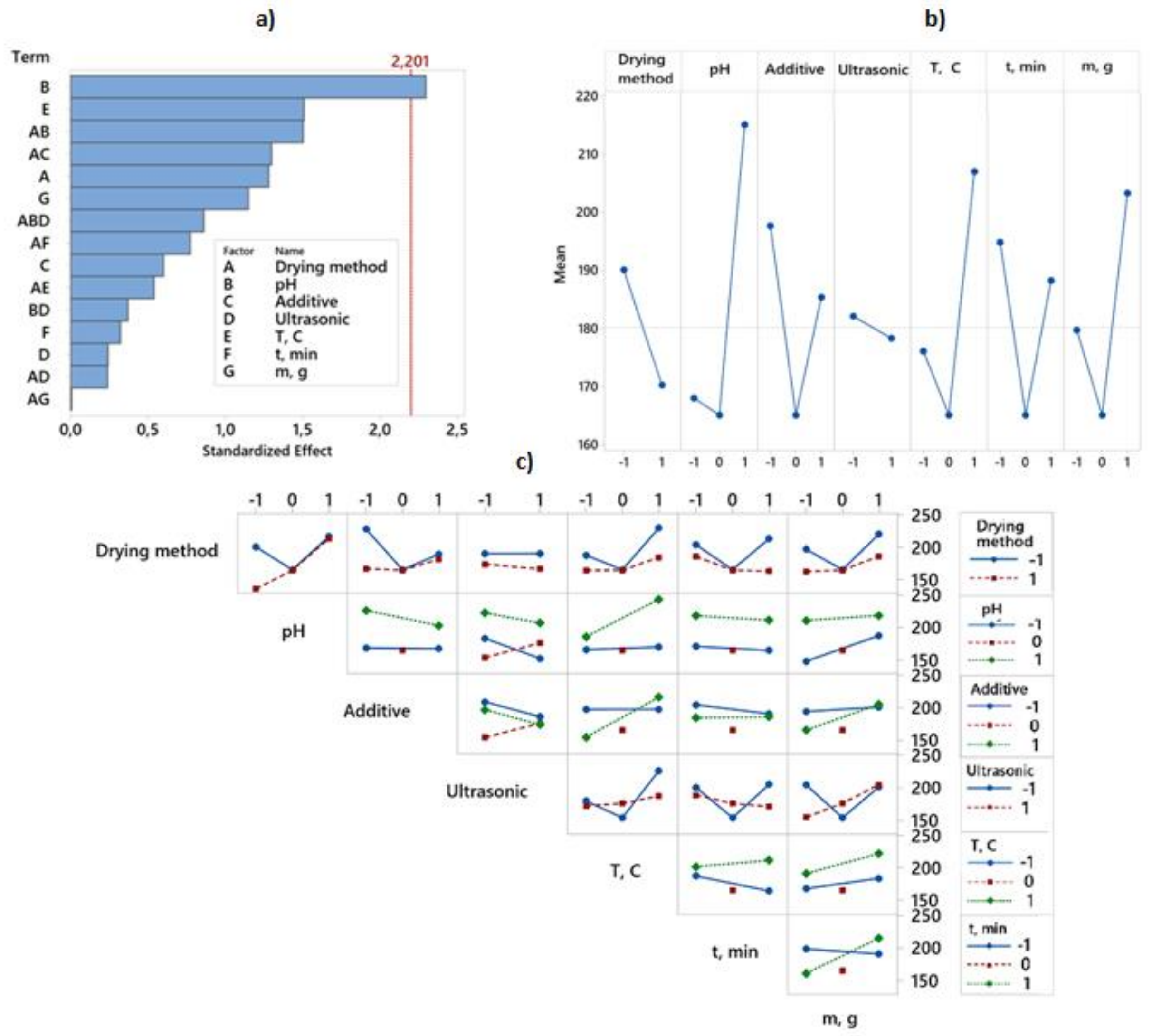

Figure S3. Texture analysis / Skin puncture force (g): (**a**) Pareto chart for standardized effect; (**b**) Main effect plot; (**c**) Interaction plot.

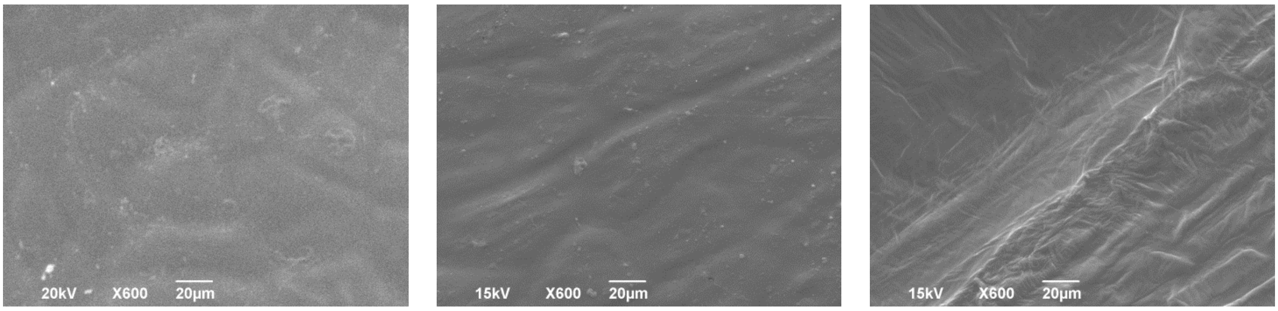

(**A**) (**B**) (**C**)

Figure S4. SEM micrographs of the dried sweet pepper: (**A**) - outer surface of sample 3 from FFD, (**B**)– outer surface of sample 24 from FFD, (**C**) – inner surface of sample 24 from FFD.

References

59. Kaur, R.; Kaur, K.; Ahluwalia, P. Effect of Drying Temperatures and Storage on Chemical and Bioactive Attributes of Dried Tomato and Sweet Pepper. *LWT - Food Sci. Technol.* **2020**, *117*, 108604, doi:10.1016/j.lwt.2019.108604.
60. Bechoff, A.; Westby, A.; Menya, G.; Tomlins, K.I. Effect of Pretreatments for Retaining Total Carotenoids in Dried and Stored Orange-Fleshed-Sweet Potato Chips. *J. Food Qual.* **2011**, *34*, 259–267, doi:10.1111/j.1745-4557.2011.00391.x.
